# Genetic screen in myeloid cells identifies TNF-α autocrine secretion as a factor increasing MDSC suppressive activity via *Nos2* up-regulation

**DOI:** 10.1038/s41598-018-31674-1

**Published:** 2018-09-07

**Authors:** Matthias Schröder, Marit Krötschel, Lena Conrad, Svenja Kerstin Naumann, Christopher Bachran, Alex Rolfe, Viktor Umansky, Laura Helming, Lee Kim Swee

**Affiliations:** 1 BioMed X Innovation Center, Heidelberg, Germany; 2 EMD Serono, Billerica, USA; 3 Skin Cancer Unit, German Cancer Research Center (DKFZ), Heidelberg, Germany; 4 Department of Dermatology, Venereology and Allergology, University Medical Center Mannheim, Ruprecht-Karls University of Heidelberg, Mannheim, Germany; 5 Merck KGaA, Darmstadt, Germany

## Abstract

The suppressive microenvironment of tumors remains one of the limiting factors for immunotherapies. In tumors, the function of effector T cells can be inhibited by cancer cells as well as myeloid cells including tumor associated macrophages and myeloid-derived suppressor cells (MDSC). A better understanding of how myeloid cells inhibit T cell function will guide the design of therapeutic strategies to increase anti-tumor responses. We have previously reported the *in vitro* differentiation of MDSC from immortalized mouse hematopoietic progenitors and characterized the impact of retinoic acid and 3-deazaneplanocin A on MDSC development and function. We describe here the effect of these compounds on MDSC transcriptome and identify genes and pathway affected by the treatment. In order to accelerate the investigation of gene function in MDSC suppressive activity, we developed protocols for CRISPR/Cas9-mediated gene editing in MDSC. Through screening of 217 genes, we found that autocrine secretion of TNF-α contributes to MDSC immunosuppressive activity through up-regulation of *Nos2*. The approach described here affords the investigation of gene function in myeloid cells such as MDSC with unprecedented ease and throughput.

## Introduction

The treatment of patients with antibodies antagonizing inhibitory molecules on the surface of T cells or tumor cells (negative immune checkpoint blockade) has already demonstrated therapeutic benefit and is now used for several indications including melanoma and non-small cell lung cancer^1^. Despite the great success of these novel immunotherapies, a sizeable proportion of patients do not respond to the checkpoint blockade^2,3^. Reported resistance mechanisms include tumor cells adaptation^4^, loss of immunogenicity^5^, up-regulation of inhibitory molecules^6^ or accumulation of immature myeloid cells^7^. Preclinical experiments have demonstrated that inhibition of myeloid cell immunosuppression can overcome resistance to checkpoint blockade^8^. In tumors, myeloid cells of different origin have been shown to inhibit T cell function including tumor-associated macrophages^9^, monocytes^10^, neutrophils^11,12^ and myeloid-derived suppressor cells (MDSC)^13^.

The development of suppressive MDSC has been shown to depend on several transcription factors including C/EBPβ^14^, HIF-1α^15^ as well as STAT proteins^16^ and their suppressive activity is mediated through cytokines, depletion of nutrients including amino acids and the production of reactive oxygen and nitrogen species^17^. In most instances, the investigation of the role of a particular gene in MDSC mediated immunosuppression has relied on the generation or availability of genetically modified animals, which can be both, cost- and time-intensive. Although myeloid cell lines can be used for genetic manipulation, they rarely reproduce more complex aspects of myeloid cell biology such as T cell priming or suppression. In order to accelerate the discovery and characterization of genes involved in e.g. myeloid cell driven immunosuppression, we established a model that reproduces MDSC biology and is amenable to genetic manipulation and screening.

We have already described the use of mouse hematopoietic progenitors immortalized through the transduction of NUP98-HOXB4 (“NUP” cells)^18^ to differentiate monocytic MDSC^19^, referred to as “NUP-MDSC” throughout this paper. The transcriptomic analysis of NUP-MDSC shown here demonstrated that features enhancing immunosuppression *in vivo* such as inflammation and HIF-1α signaling take place in a cell intrinsic manner *in vitro*. In order to investigate gene function in a straightforward manner, we established NUP cells from Cas9 knock-in mice^20^. We developed a protocol for efficient gene editing upon transduction with gRNAs and used this system to investigate the role of 217 genes in NUP-MDSC suppressive activity. Our results indicate that TNF-α secretion by NUP-MDSC increases their T cell suppressive activity by up-regulating *Nos2*.

## Results

### Transcriptomic analysis reveals the activation of innate sensing pathways and HIF-1α signaling in NUP-MDSC in absence of external inflammatory stimuli or hypoxia

**Figure 1:**
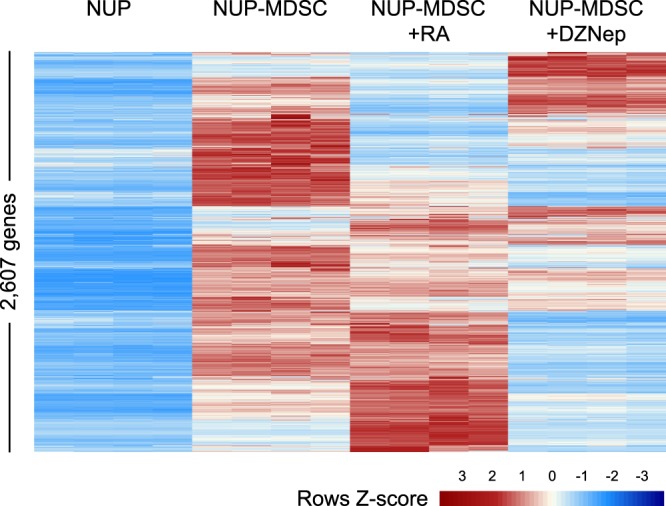
Analysis of differential gene expression upon NUP-MDSC differentiation and upon treatment with retinoic acid or 3-deazaneplanocin A. NUP cells were differentiated *in vitro* with GM-CSF/IL-6 for 4 days in the presence or absence of retinoic acid (RA) or 3-deazaneplanocin A (DZNep) or kept undifferentiated (4 replicates per condition). Gene transcription was analyzed by Next Generation RNA sequencing. The heatmap shows relative expression in all samples of 2.607 genes up-regulated in NUP-MDSC vs NUP cells (fold change ≥ 2, adj P ≤ 0.05).

We have previously described the differentiation of NUP cells into MDSC (NUP-MDSC) by treatment with GM-CSF and IL-6^19^. We compared these NUP-MDSC with MDSC differentiated from fresh mouse bone marrow using established protocols^14^ and demonstrated that NUP-MDSC contained ~90% of CD11b^+^Ly-6C^+^Ly-6G^−^ monocytic cells which displayed STATs signaling, the production of reactive oxygen species (ROS) and reactive nitrogen species (RNS), expression of IL-4Rα, down-regulation of IRF8, arginase activity as well as the expression of S100A8/9 proteins^19^. Importantly, NUP-MDSC and bone marrow-derived MDSC had similar suppressive activity. Furthermore, we demonstrated that retinoic acid (RA) and 3-deazaneplanocin A (DZNep) alter the differentiation of suppressive cells as well as their functions^19^. In order to uncover potential new mechanisms, by which MDSC suppress T cells and to better understand how RA and DZNep inhibit the function of MDSC, we analyzed the transcriptome of undifferentiated NUP cells and NUP-MDSC treated with DMSO, RA or DZNep. Differential gene expression analysis revealed that a total of 2,607 genes were up-regulated in NUP-MDSC vs. NUP cells (fold change ≥ 2, adjusted P ≤ 0.05, Fig. 1). RA and DZNep treatment resulted in apparent down-regulation of a considerable fraction of genes up-regulated in NUP-MDSC (Fig. 1). Ingenuity pathway analysis revealed that genes involved in myeloid cell development, cytokine signaling and HIF-1α signaling were up-regulated in NUP-MDSC (Table 1). Several inflammatory molecules including cytokines (TNF-α, IL-1β) and Toll-Like-Receptor (TLR) agonists (LPS, poly I:C) were found among the most significant potential upstream regulators (NUP-MDSC vs. NUP, Table 2). Treatment of NUP-MDSC with RA induced a down-regulation of genes regulated by TLR agonists and NF-κB (Table 3), whereas DZNep treatment significantly reduced HIF-1α signature (Table 4) as already suggested by our earlier study^19^. Altogether, our analysis shows that (1) inflammatory and HIF-1α signaling are up-regulated in NUP-MDSC and (2) gene up-regulation is counteracted by the treatment of RA and DZNep.

**Table 1:** Top 20 up-regulated pathway (MDSC vs NUP) (Ingenuity pathway analysis).

Ingenuity Canonical Pathways	−log(p-value)	Ratio	z-score
Hepatic Fibrosis/Hepatic Stellate Cell Activation	1.34E01	3.13E-01	NaN
Granulocyte Adhesion and Diapedesis	9.51E00	2.77E-01	NaN
Agranulocyte Adhesion and Diapedesis	9.43E00	2.7E-01	NaN
Altered T Cell and B Cell Signaling in Rheumatoid Arthritis	9.3E00	3.6E-01	NaN
Dendritic Cell Maturation	7.79E00	2.59E-01	4.217
Colorectal Cancer Metastasis Signaling	7.62E00	2.33E-01	3.434
Graft-versus-Host Disease Signaling	6.28E00	3.91E-01	NaN
MSP-RON Signaling Pathway	6.28E00	3.91E-01	NaN
IL-10 Signaling	5.86E00	3.24E-01	NaN
Communication between Innate/Adaptive Immune Cells	5.85E00	2.92E-01	NaN
IL-8 Signaling	5.8E00	2.3E-01	4.003
HIF1α Signaling	5.74E00	2.77E-01	NaN
TREM1 Signaling	5.65E00	3.07E-01	3.128
Role of Tissue Factor in Cancer	5.44E00	2.64E-01	NaN
Role of Macrophages, Fibroblasts and Endothelial Cells in Rheumatoid Arthritis	5.37E00	1.97E-01	NaN
Glioma Invasiveness Signaling	5.37E00	3.33E-01	2.524
STAT3 Pathway	5.29E00	3.01E-01	0.853
Caveolar-mediated Endocytosis Signaling	4.94E00	2.96E-01	NaN
ILK Signaling	4.89E00	2.16E-01	3.087
Role of Osteoblasts, Osteoclasts and Chondrocytes in RA	4.87E00	2.06E-01	NaN

**Table 2:** Top 20 upstream regulators (MDSC vs NUP) (Ingenuity pathway analysis).

Upstream Regulator	Exp Log Ratio	z-score	p-value of overlap
lipopolysaccharide		9.716	1.17E-65
phorbol myristate acetate		7.956	1.30E-36
IFNG		7.373	3.36E-49
TNFSF11	2.616	6.989	1.65E-29
TNF	1.625	6.972	8.47E-55
poly rI:rC-RNA		6.682	5.43E-18
IL1B	3.579	6.411	9.03E-40
TGM2	2.087	6.221	3.81E-13
TLR9	−0.034	6.197	7.45E-14
mir-223		6.122	2.62E-27
E. coli B5 lipopolysaccharide		6.116	2.72E-17
TLR3	1.852	6.092	1.06E-13
bleomycin		6.077	5.32E-23
IL5	−0.811	5.934	3.06E-18
E. coli B4 lipopolysaccharide		5.916	1.85E-17
Salmonella enterica serotype abortus equi LPS		5.757	1.19E-21
CSF2		5.756	4.61E-34
NFkB (complex)		5.692	4.83E-27
tretinoin		5.640	1.20E-36

**Table 3:** Top 20 down-regulated upstream regulators (RA vs DMSO in MDSC) (Ingenuity pathway analysis).

Upstream Regulator	Exp Log Ratio	z-score	p-value of overlap
TNFSF11	0.065	−3.896	1.53E-17
FOXO1	0.095	−3.679	4.41E-05
TLR9	1.129	−3.601	1.73E-12
IL2		−3.478	6.87E-13
thapsigargin		−3.397	2.46E-09
IL18	0.022	−3.395	4.23E-09
TRPV4	0.524	−3.286	4.19E-08
UCP1		−3.197	1.04E-04
salmonella minnesota R595 LPS		−3.111	1.58E-09
DCN		−3.096	7.19E-08
MAP3K14	0.719	−3.058	8.17E-06
paclitaxel		−3.052	1.15E-09
NOD2	−0.313	−3.048	1.86E-07
NFkB (complex)		−3.015	5.31E-19
resiquimod		−2.954	6.71E-10
IL12A		−2.952	5.63E-03
CD40LG	1.707	−2.946	1.12E-09
E. coli lipopolysaccharide		−2.945	9.21E-04
imiquimod		−2.920	6.14E-02
RELA	−0.150	−2.892	3.94E-10

**Table 4:** Top 20 down-regulated upstream regulators (DZNep vs DMSO in MDSC) (Ingenuity pathway analysis).

Upstream Regulator	Exp Log Ratio	z-score	p-value of overlap
STAT4	0.137	−5.677	4.28E-16
phorbol myristate acetate		−4.475	4.94E-21
TRIM24	−0.738	−4.157	5.89E-11
MKL2	−0.328	−4.025	3.29E-10
TNF	−0.237	−3.871	1.63E-21
TREM1	−1.583	−3.683	1.67E-10
MKL1	0.317	−3.674	3.37E-08
HIF1A	−0.953	−3.652	2.27E-11
SOCS1	0.017	−3.603	3.57E-07
ACKR2	−0.066	−3.578	5.08E-11
SMAD4	−0.614	−3.494	3.13E-03
PDGF BB		−3.395	8.04E-09
SRF	0.837	−3.328	2.55E-05
IL5	0.164	−3.308	1.40E-13
PTGER4	0.634	−3.298	1.48E-15
ID3	1.402	−3.241	4.14E-07
STAT3	−0.373	−3.239	6.91E-16
miR-3183 (and other miRNAs w/seed CCUCUCU)		−3.196	7.44E-04
TNFRSF8	1.012	−3.162	8.70E-02
CD38	−0.914	−3.156	6.41E-10

### CRISPR/Cas9-mediated gene-editing in NUP-MDSC

**Figure 2:**
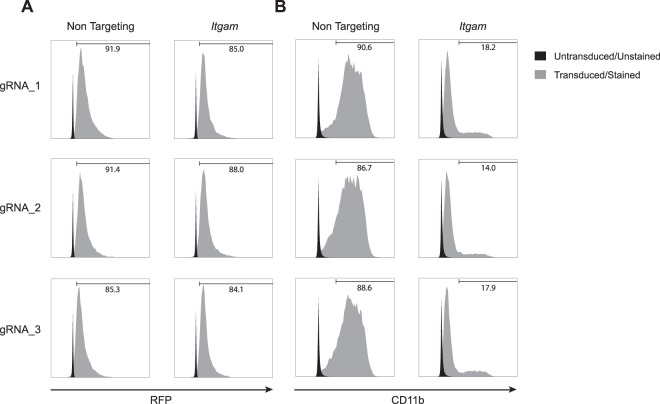
Gene editing in NUP^Cas9^ cells. NUP^Cas9^ cells were transduced with constructs encoding for a puromycin resistance gene, RFP and a gRNA and selected with puromycin. (**A**) Histograms show RFP expression in control NUP-MDSC^Cas9^ cells (black) or NUP-MDSC^Cas9^ cells transduced with a construct encoding for non-targeting or *Itgam*-specific gRNA (gray). (**B**) Histograms show CD11b expression in control NUP-MDSC^Cas9^ (black) or NUP-MDSC^Cas9^ cells transduced with a construct encoding for non-targeting or *Itgam*-specific gRNA’s (gray).

The characterization of gene function in MDSC would benefit from approaches that do not rely on the generation of genetically modified animals. In order to perform gene-editing in NUP cells, we established NUP cells from Cas9 knock-in animals^20^ (NUP^Cas9^). We then transduced NUP^Cas9^ cells with a vector encoding for a puromycin resistance gene, Red Fluorescence Protein (RFP) and a gRNA targeting sequence. Our results show that upon transduction with 3 non-targeting or *Itgam* (CD11b)-specific gRNAs and puromycin selection, 85–90% of the cells expressed RFP (Fig. 2A). Most importantly, specific targeting of *Itgam* resulted in 80–85% reduction in CD11b expression in NUP-MDSC^Cas9^ (Fig. 2B). Therefore, transduction of NUP^Cas9^ cells with gRNA followed by antibiotic selection enables efficient gene editing resulting in loss of corresponding protein expression.

### Genetic screen of NUP-MDSC^Cas9^ cells suppressive activity

**Figure 3:**
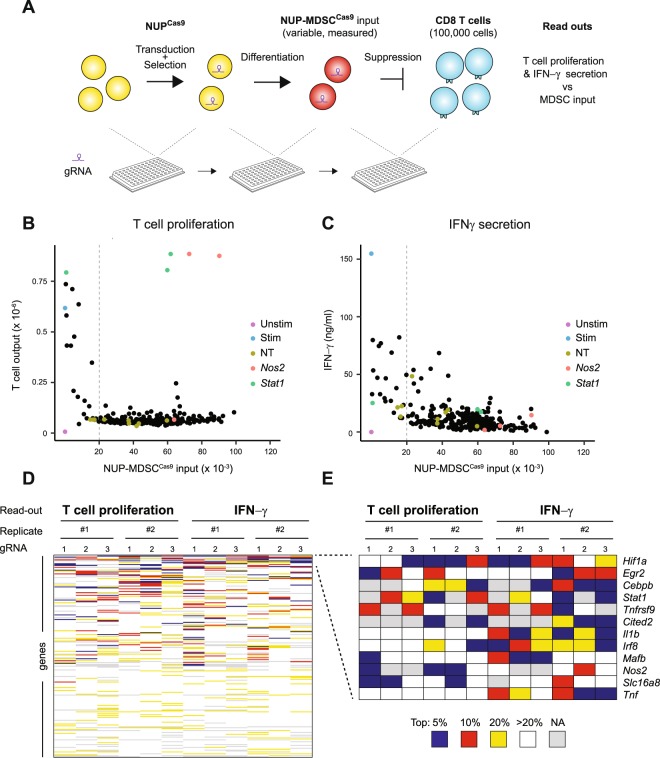
Genetic screen of suppression activity. (**A**) Overview of the screen (1) NUP^Cas9^ cells were transduced with gRNA constructs and selected with puromycin (2) NUP^Cas9^ were differentiated into NUP-MDSC^Cas9^ (3) NUP-MDSC^Cas9^ numbers were recorded and cells co-cultured with stimulated CD8 T cells. After 3 days, T cells numbers and IFNγ was measured and compared with NUP-MDSC^Cas9^ input. (**B**,**C**) NUP-MDSC^Cas9^ input vs T cells numbers or IFN-γ after 3 days of co-culture. Controls (unstimulated T cells, stimulated T cells, stimulated T cells co-cultured with NUP-MDSC^Cas9^ transduced with non-targeting gRNAs) as well as selected genes (stimulated T cells co-cultured with NUP-MDSC^Cas9^ transduced with gRNA specific for *Nos2* or *Stat1*) are highlighted. (**D**) Overview of the screen. The heatmap shows gRNAs rank (per plate) based on their effect on NUP-MDSC^Cas9^ suppressive activity for each gRNA for 2 independent experiments and 2 different readouts. (**E**) Genes for which at least 4 gRNAs in total (3 gRNAs per gene, 2 independent experiments, 2 read outs) rank in the top 10%.

In order to investigate the function of hundreds of genes in parallel in the context of immunosuppression, we designed protocols compatible with gene editing, MDSC differentiation and T cell suppression in a medium throughput format. These protocols rely on the cloning, transduction, differentiation and suppression in a 96-well plate format (Fig. 3A). We observed an expected well-to-well variation in NUP-MDSC^Cas9^ numbers after differentiation. Immunosuppression is a function of the ratio between T cells and MDSC. Therefore, in order to perform the suppression screen while taking into account different NUP-MDSC^Cas9^ input in the different wells, we stimulated a constant number of CD8 T cells together with a variable but known number of gene-edited NUP-MDSC^Cas9^ monitored by high throughput flow cytometry. After 3 days of stimulation, T cell proliferation and IFN-γ secretion was measured and plotted with the input of NUP-MDSC^Cas9^ present in each well. Based on our transcriptomic analysis and on literature, we selected 217 genes to be tested in this suppression assay (Supplementary Table 1). For each gene, we selected 3 gRNAs, which were tested individually in 2 independent experiments (>1,400 wells acquired in total). Each independent screen was performed in experiment batches of 2–4 plates each. An example of such experiments is shown in Figure 3B,C. Scatter plots in Figure 3B,C show MDSC input vs. T cell proliferation or IFN-γ production for each gRNA or control (unstimulated T cells, stimulated T cells alone). Data showed clear suppression of T cell proliferation and IFN-γ secretion up to an input of 20,000 NUP-MDSC^Cas9^ (T:MDSC ratio of 5:1), below which little or no suppression could be observed. Figure 3B,C highlight genes, which editing induced a loss in NUP-MDSC^Cas9^ suppressive activity such as *Nos2* and *Stat1*. Detailed analysis revealed some variations, both in the mean and spread of data between plates of a particular experiment, presumably due to differences in plate handling during data acquisition (HT-flow cytometry, ELISA etc). Therefore, for overall analysis, we assigned ranks for each gRNA per plate and readout according to its effect on MDSC suppressive activity. Data points, for which the NUP-MDSC^Cas9^ input was lower than 20,000 cells, were excluded from the ranking. We then ranked genes according to the number of gRNAs amongst the top 10% (Fig. 3D). Top ranking genes included genes known to contribute to MDSC suppressive activity (*Hif1a*, *Cebpb*, *Stat1*, *Nos2)* as well as inflammatory cytokines (*Tnf*, *Il1b*) (Fig. 3E).

### Autocrine TNF-α secretion increases NUP-MDSC suppressive activity

**Figure 4:**
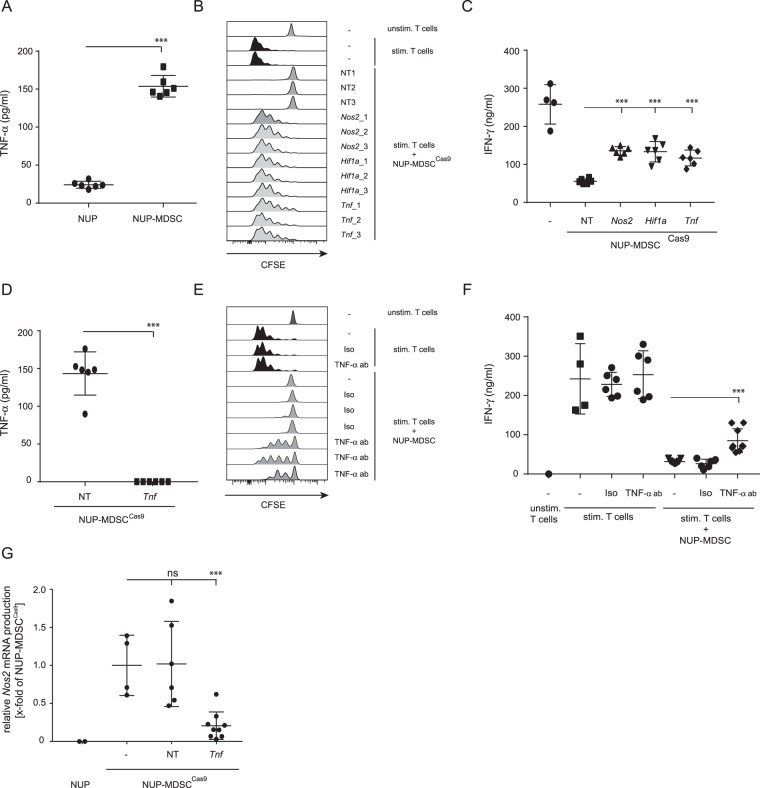
Autocrine TNF-α secretion and impact on MDSC suppressive activity. (**A**) TNF-α concentration in the supernatant of NUP or NUP-MDSC cells. (**B**,**C**) CFSE-labelled CD8 T cells were stimulated alone or in the presence of NUP-MDSC^Cas9^ transduced with non-targeting gRNAs or gRNAs targeting *Nos2*, *Hif1a* or *Tnf*. (**B**) Histograms show CFSE dilution on CD8 T cells after 3 days of stimulation. (**C**) IFNγ concentration in the supernatant after 3 days of stimulation. Summary of 2 independent experiments (3 gRNAs/gene). (**D**) TNF-α concentration in the supernatant of NUP-MDSC^Cas9^ cells transduced with non-targeting gRNAs (NT) or gRNAs specific for *Tnf*. Summary of 2 independent experiments (3 gRNAs/gene). (**E** and **F**) CFSE-labeled CD8 T cells were stimulated alone or in the presence of NUP-MDSC together with a neutralizing antibody against TNF-α or an isotype control. (**E**) Histograms show CFSE level on CD8 T cells after 3 days of stimulation. (**F**) IFN-γ concentration in the supernatant after 3 days of stimulation. Summary of 2 independent experiments. (**G**) Relative *Nos2* expression in NUP cells, NUP-MDSC^Cas9^ and NUP-MDSC^Cas9^ transduced with non-targeting gRNAs or gRNAs targeting *Tnf*. Summary of 3 independent experiments (3 gRNAs/gene). ***P < 0.001, Student T-test.

The marked up-regulation of inflammatory pathways and cytokines (TNF-α, IL-1β) in NUP-MDSC vs. NUP cells (Table 2) suggests that these events may initiate a gene expression program contributing to suppressive activity. We measured TNF-α concentration in the supernatant of NUP cells or NUP-MDSC and observed a marked increase in the TNF-α secretion upon differentiation (Fig. 4A). However, IL-1β was undetectable. To validate the role of TNF-α secretion by NUP-MDSC in their suppressive activity, we established *Tnf*-edited NUP-MDSC^Cas9^ and compared their suppressive activity with NUP-MDSC^Cas9^ transduced with non-targeting gRNAs, or gRNAs specific for *Hif1a* or *Nos2*. Our results confirmed that *Tnf*-edited as well as *Hif1a*- and *Nos2*-edited NUP-MDSC^Cas9^ partially lose suppressive activity (Fig. 4B,C). As expected, TNF-α could not be measured in *Tnf*-edited NUP-MDSC^Cas9^ (Fig. 4D). Furthermore, inhibition of TNF-α activity using neutralizing antibody but not isotype control partially inhibited NUP-MDSC activity (Fig. 4E,F). Given the role of *Nos2* in MDSC suppressive activity in our experiments and based on reports showing up-regulation of *Nos2* expression by *Tnf *^21^, we postulated that autocrine secretion of TNF-α would increase *Nos2* expression and therefore MDSC suppressive function. To test this hypothesis, we measured *Nos2* mRNA level in NUP-MDSC^Cas9^ transduced with non-targeting gRNA compared to *Tnf*-edited NUP-MDSC^Cas9^ and demonstrated a decreased *Nos2* expression in *Tnf*-edited cells (Fig. 4G). Altogether, GM-CSF/IL-6 differentiation of NUP cells into NUP-MDSC induced the autocrine secretion of TNF-α, which increased suppressive activity through up-regulation of *Nos2*.

## Discussion

In the present study we have analyzed global gene expression upon differentiation of NUP cells into MDSC. Interestingly, the analysis of pathways and upstream regulators revealed up-regulation of genes associated with HIF-1α signaling and inflammation, including TLR agonists and TNF-α. Cells were differentiated in normoxic conditions and in absence of treatment other than GM-CSF and IL-6 suggesting that HIF-1α and inflammatory signaling is due to cell-intrinsic mechanisms rather than to external cues. HIF-1α signaling and inflammation have been shown to contribute to MDSC immunosuppressive activity. Indeed, deficiency in HIF-1α results in decreased suppressive activity and increased anti-tumor response^15^. Our own results confirm that *Hif1a*-edited NUP-MDSC^Cas9^ have decreased activity however these data reveal a potential function of HIF-1α that is uncoupled from hypoxia. Furthermore, we have also shown that DZNep inhibited the differentiation of immunosuppressive MDSC and demonstrated that DZNep-treated MDSC failed to up-regulate HIF-1α upon hypoxia^19^. Strikingly, pathway analysis of genes affected by RA and DZNep treatment showed that compounds counteracted inflammatory (TLR agonists, NF-κB) and HIF-1α-associated signatures respectively.

Inflammation has been shown to contribute to immunosuppression^22–26^. For example, members of the S100 protein family have been shown to induce both the migration of myeloid cells into the tumor as well as the amplification of inflammation through the binding to RAGE and TLR4 receptors^22–24^. Pathogen-derived TLR agonists have been shown to induce accumulation of MDSC or to increase their activity^25,26^. Some studies, however, have demonstrated differentiation of mature myeloid cells promoting anti-tumor response upon treatment with TLR agonists^27,28^. Altogether, the impact of TLR agonists on anti-tumor response seems to be dependent on the cellular context^29,30^. The role of inflammatory cytokines such as TNF-α in MDSC biology was previously investigated in a model of chronic inflammation where authors reported the role of TNF-α in blocking myeloid cells differentiation and increasing their immunosuppressive activity^21^. In the context of cancer, TNF-α neutralization was shown to delay tumor growth and decrease an MDSC accumulation^31^. Data presented here uncover the role of TNF-α as an autocrine factor increasing MDSC activity. Indeed, we observed the secretion of TNF-α upon the MDSC differentiation as well as a TNF-α signaling signature in NUP-MDSC. Importantly, we demonstrated that TNF-α secretion increased MDSC activity via up-regulation of *Nos2*. However, the cause(s) of this inflammatory program and TNF-α secretion in NUP-MDSC remains unknown. The secretion of TNF-α by MDSC could be induced directly by GM-CSF. Indeed, GM-CSF is an inflammatory cytokine^32^ that has been shown to be secreted by human tumor cells and cancers and to contribute to MDSC development and suppressive activity^33–35^. Alternatively, GM-CSF could induce inflammation indirectly through the up-regulation or release of cytokines, endogenous TLR agonists and danger-associated molecular patterns (e.g. S100 protein, HMGB1, heat shock proteins)^36^.

Altogether, inhibiting inflammation and/or positive feedback loop increasing inflammation could offer therapeutic opportunities to decrease an immunosuppression and increase the anti-tumor response.

Finally, our study describes a novel scalable approach to investigate the function of genes in myeloid cell development or function in a medium throughput manner. It is compatible with investigations of other myeloid cell subsets (macrophages, dendritic cells) or functions (host-pathogen interaction, T cell priming) and can accelerate research without the need of animal husbandry and experimentation.

## Materials and Methods

### Mice

C57BL/6j mice and Gt(ROSA)26Sortm1.1(CAG-cas9-EGFP)Fezh/J (Cas9 knock-in)^20^ were maintained under specific pathogen–free conditions in the animal facility of the University of Heidelberg. The mice were sacrificed for T cell and bone marrow isolation only and not used for other animal experiments, hence making the need for an official ethics review unnecessary as the mice do not suffer (based on §1, §4 passage 3 TierSchG (4.7.2013) and TierSchVersV (12.08.2013) §2, annex 1, annex 2).

### Generation of NUP98/HOXB4 cells

To generate NUP98/HOXB4 cells we used freshly isolated bone marrow cells of C57BL/6j or Cas9 knock-in mice. The bone marrow cells were treated with ACK lysis buffer and subsequently depleted for CD19^+^ and Gr1^+^ cells with magnetic cell isolation and cell separation (MACS). The remaining cells were cultured for 3 days in NUP medium (see below) before using them for retroviral transduction.

The expression plasmids pSTITCH-GFP (pST-GFP), pHit60 [murine leukemia virus (MLV)-Gag/Pol], pHit123 [ecotropic MLV envelope], as well as MLV-A [amphotropic MLV envelope] were kindly provided by Reno Debets (Department of Medical Oncology, Erasmus MC, Rotterdam, The Netherlands)^37^. To generate pST-NUP98-HOXB4-IRES-Hyg, cDNA encoding the fusion protein NUP98-HoxB4 was amplified from pMyc-NUP98-HOXB4-IP (kind gift from Klaus Karjalainen & Antonius Rolink) and transferred into pST-GFP. Simultaneously, GFP was replaced by the hygromycin resistance gene using the Gibson assembly cloning strategy (New England BioLabs, Ipswich, USA).

MLV-based vector production was performed as described previously^19^. Virus containing supernatant was harvested after 72 h and either used immediately for transduction or stored at −80 °C until further use.

Retroviral transduction was performed as follows: 24-well plates were coated with 16 µg/mL RetroNectin (Takara Bio Europe/Clontech, #T100B) for 2 h at 37 °C. Subsequently, 2 ml of virus containing medium was spun on the plates at 3200 g for 1 h. Bone marrow progenitors were resuspended at 1 × 10^7^ cells/mL in NUP growth medium, mixed gently with 4 µg/mL polybrene (Sigma Aldrich, #107689) and 2 × 10^6^ cells were added per 24-well. Spin-inoculation was performed at 650 g for 1 h at room temperature. 2 h after transduction the cells were washed once and resuspended in NUP growth medium at a density of 0.5 × 10^6^ cells/mL. Antibiotic selection with 300 µg/mL hygromycin B (Merck KGaA, #400050) was performed from d2 to d5 after transduction.

### Cultivation of NUP98/HOXB4 cells

NUP98/HOXB4 cells were cultivated in RPMI1640 medium (Life Technologies, 21875-034) containing 10% FBS (Life Technologies, #10270106) 100 U/mL penicillin (Life Technologies, #15140122) 100 µg/mL streptomycin (Life Technologies, #15140122), 1 mM sodium pyruvate (Life Technologies, #11360070), 50 µM β-mercaptoethanol (Life Technologies, 31350-010), 1x non-essential amino acids (Life Technologies, 11140-035), 20 ng/ml IL-6 (Biolegend, #575708) and 10 ng/ml SCF (Biolegend, #579708) in tissue culture flasks (37 °C, 5% CO_2_). Every two or three days, the NUP cells were split to a concentration of 0.2–0.3 × 10^6^ cells/mL and cultured for up to three weeks. To induce MDSC differentiation the NUP cells were pelleted and resuspended in RPMI1640 medium (10% FBS, 100 U/mL penicillin, 100 µg/mL streptomycin, 1 mM sodium pyruvate, 50 µM β-mercaptoethanol, 1x non-essential amino acids) containing 20 ng/mL IL-6 and 20 ng/mL GM-CSF (Biolegend, #576304). The NUP cells were differentiated into MDSCs for four days and then used for subsequent experiments. To investigate the effects of inhibitors on MDSC some NUP cells have been co-cultured with 0.1% DMSO, 1 µM retinoic acid (Sigma Aldrich, R2625) or 0.1 µM 3-Deazaneplanocin (Cayman Chemicals, 13828) during the four days of differentiation.

### CRISPR screening

#### Cloning strategy

For the CRISPR screening we used Cas9 knock-in NUP cells that constantly express Cas9 protein. To induce the CRISPR process we introduced separate gRNAs via lentiviral transduction. We used a predesigned list from Doench *et al*.^38^ as basis for our cloning strategy and picked three different primer per gene. We added a CACCG (5′-end) to the forward primer and a C (3′-end) to the reverse primer and ordered the synthesis (Eurofins Genomics, Ebersberg). Forward and reverse primer for each gene was then mixed and phosphorylated with T4 polynucleotide kinase (New England Biolabs, M0201L) for 30 min at 37 °C, followed by a 5 min deactivation at 95 °C and a cool down gradient to 25 °C with 4 °C/min. For the sub-cloning process the primer were diluted to 0.5 µM. The plasmid pRSI12 U6 with a gRNA scaffold (Cellecta, DVSHC-NT-PS) was linearized with BbsI (New England Biolabs, R0539L) and dephosphorylated with shrimp alkaline phosphatase (New England Biolabs, M0371S). The sample was run on an agarose gel, eluted from the gel and DNA concentration determined. For the ligation we incubated 1 µl phosphorylated oligos (0.5 µM) with 50 ng BbsI-linearized plasmid, 1 µl 10 × T4 DNA ligase (New England Biolabs, B0202S), 0.5 µl T4 DNA ligase (New England Biolabs, M0202L) and 7 µl water for 1 h at room temperature and 10 min at 65 °C. The ligation mix was then used for transformation of Stbl3 cells (Thermo Fisher Scientific GmbH, C737303). 96-well plates, containing LB-media with ampicillin, were then inoculated with the transformed Stbl3 cells and shipped for sequencing (GATC Biotech, Konstanz). The plasmid DNA (pRSI12 U6-gRNA) was sent back and stored at −20 °C until needed.

#### Virus production

Cell culture grade 96-well plates were coated with 70 µg/ml collagen in PBS for 1 h at 37 °C and then washed with PBS once, before seeding 1.5 × 10^4^ Hek293 cells in DMEM supplemented with 10% FBS, 100 U/mL penicillin, 100 µg/mL streptomycin, 1 mM sodium pyruvate, 50 µM β-mercaptoethanol and 1x non-essential amino acids. One day later the Hek293 cells got transfected using JetPEI (VWR International GmbH, 101-10 N), containing 0.13 µg psPax2 (Cellecta, CPCP-K2A), 0.07 µg pMD2.G (Cellecta, CPCP-K2A) and 0.3 µg of pRSI12 U6-gRNA. After 5 h the transfection mix was aspirated and substituted with 200 µl DMEM medium with all supplements. 72 h later the virus-containing supernatant was harvested, aliquoted on two 96-well plates and stored at −80 °C until needed.

#### Transduction of Cas9 knock-in NUP cells

Cell culture grade 96-well plates were coated with 16 µg/ml Retronectin (Takara Bio Europe/Clontech, #T100B) for 2 h at 37 °C and then washed with PBS once before adding 100 µl of the virus-containing supernatant. After a 1 h centrifugation at 3200 g at 18 °C we added 2 × 10^5^ Cas9 knock-in NUP cells per well and 4 µg/ml polybrene (Sigma Aldrich, #107689) and centrifuged for 1 h at 30 °C with 650 g. Subsequently the plate was incubated at 37 °C and 5% CO_2_ for 4 h and then the virus-containing supernatant was aspirated and substituted with normal NUP cell growth medium. Antibiotic selection was started 48 h later with 1 µg/ml puromycin (VWR International GmbH, J593) for 2 days. The surviving NUP cells were then cultured for another 48 h in NUP cell growth medium before using them for the differentiation process.

#### MDSC differentiation and characterization

For the differentiation process in 96-well plates we did count the cell number per well with a BD FACSCanto RUO Special Order System (BD Biosciences, Heidelberg). For the differentiation we seeded an average of 2 × 10^4^ CRISPRed NUP cells per well, based on the mean cell count of all 96-wells, in RPMI1640 medium (10% FBS, 100 U/mL penicillin, 100 µg/mL streptomycin, 1 mM sodium pyruvate, 50 µM β-mercaptoethanol, 1x non-essential amino acids) containing 20 ng/mL IL-6 and 20 ng/mL GM-CSF. After 4 days we analyzed the cell count, the expression of Gr1, CD11b, Ly6C and Ly6G surface marker and RFP fluorescence as an indicator for properly transduced cells. Wells with MDSC that were less than 80% RFP-positive were excluded from the final analysis. For the suppression assay we seeded an average of 5 × 10^4^ CRISPRed MDSC per well, based on the mean cell count of all 96-wells, aiming for a 1:2 ratio with 1 × 10^5^ CD8^+^ T cells. If the average MDSC-input was lower than 2 × 10^4^ (<1:5 ratio) the results were excluded from the final analysis. This threshold was set based on the finding that the NUP cell derived MDSC are not able to suppress T cells below a 1:5 ratio^19^.

### T cell isolation and suppression assay (MIATA-compliant)

#### The Sample

WT mice were transported from the animal facility to our laboratory and sacrificed immediately. Spleen and lymph nodes of WT mice were isolated, mashed through a 40 µm cell strainer (NeoLab, ST292712340) and incubated 5 min with ACK-lysing buffer (Life Technologies, A10492-01) at room temperature. The process was stopped with MACS buffer (1× PBS, 2% FBS and 2 mM EDTA) and after a centrifugation step the cells were counted and resuspended in MACS buffer for subsequent MACS CD8^+^ T cell isolation (Miltenyi Biotech, 130-104-075) according the manufacturer’s instructions. The whole process from organ isolation to single cell suspension took approximately 1.5 h.

None of the cells were stored/frozen and reused later.

An aliquot of the cells was stained with trypan blue (Life Technologies, #15250-061) to discriminate between alive and dead cells and counted with a Neubauer improved chamber using a microscope (Leica, Wetzlar). The median cell yield per mouse (splenocytes and lymphnodes) was 1.2 × 10^8^ and the CD8^+^ T cell yield ~9 × 10^6^ per mouse. Cell viability was >95% and the purity of the CD8^+^ T cell fraction was >90% (detected with FACS). Before using the cells for the assay they were incubated with 1 µM CFSE (Biolegend, #423801) in PBS for 5 min at 37 °C. Afterwards the cells were pelleted and resupended in assay medium (see below).

#### The Assay

The assay was performed in RPMI1640 medium (Life Technologies, 21875-034) containing 10% FBS (Life Technologies, #10270106) 100 U/mL penicillin (Life Technologies, #15140122) 100 µg/mL streptomycin (Life Technologies, #15140122), 1 mM sodium pyruvate (Life Technologies, #11360070), 50 µM β-mercaptoethanol (Life Technologies, 31350-010) and 1x non-essential amino acids (Life Technologies, 11140-035). The serum used for all experiments was from the same batch and was constantly monitored by the provider (Life Technologies).

Before adding the freshly isolated CD8^+^ T cells into the assay, flat-bottom 96-wells were coated with 2 μg/mL anti-CD3 and 2 μg/mL anti-CD28 antibodies (BioLegend #100302 and #102102) in 50 μL PBS per well for 30 minutes at 37 °C. After washing the wells with PBS, 1 × 10^5^ CFSE labelled CD8^+^ T cells were added to each well. Recombinant mouse IL-2 (Biolegend, #575402) was added to induce proliferation at a final concentration of 5 ng/ml. 0.5 × 10^5^ NUP cells or different amounts of freshly generated MDSC (0.2–1 × 10^5^) were added. T cells without mIL-2 and without CD3/28 stimulation were used as negative control and stimulated T cells without addition of other cells as positive control. NUP cells were added in each replicate to demonstrate that there is no suppressive activity prior to the differentiation process into MDSC. MDSCs, transduced with non-targeting gRNA (NT), were added in each replicate to demonstrate that there is no effect of the transduction process on suppressive activity.

The Ultra-LEAF^TM^-purified anti-Tnf-α antibody was used at a concentration of 5 µg/ml to deplete produced Tnf-α from the supernatant (Biolegend, #506331). After a 72 h incubation at 37 °C and 5% CO_2_, the cells were transferred into a 96-well V-bottom plate and pelleted. The supernatant was saved for subsequent measurement of cytokines while the cells were stained with CD8α-eFluor450 (eBioscience, #48-0081) to determine proliferation by measuring the CFSE signal via FACS.

#### Data Acquisition

Samples were acquired using the FACSAria IIu (BD Biosciences, Heidelberg) and FACS diva software (TreeStar). CS&T beads (BD Biosciences, 655051) were used to set up the Photo Multiplier Tube (PMT) voltages prior to measuring samples. Samples were compensated using single stained, CFSE negative T cells and samples. The unstimulated T cells were used as reference for cells that did not proliferate.

#### Raw data

As described, stimulated T cells were used as positive control, representing the status of maximal proliferation and maximal IFNγ and TNFα production. For unstimulated T cells there was no background reactivity detectable, neither for proliferation nor for cytokine production. Raw data can be provided per request. Suppressive activity due to MDSC co-culture was pre-defined by reduced T cell proliferation and/or significantly reduced cytokine production. Data analysis was performed using the software Microsoft Office Excel as well as GraphPad Prism. Statistical analysis of results was done by performing unpaired, two-tailed t-test with Welch’s correction using GraphPad Prism. Significance levels were defined as *p < 0.05, **p < 0.01 and ***p < 0.001.

#### General Lab Operation

These studies were conducted in a laboratory that operates under exploratory research principles. The study was performed using investigative and established laboratory protocols and were performed using investigative and validated assays.

### RNA isolation, cDNA synthesis and quality analysis

For RNA isolation 5 × 10^6^ cells were washed in PBS and resuspended in 300 µL Trizol (LifeTechnologies, #15596026). The RNA was isolated according the manufacturer’s instructions. The RNA concentration was determined using a Qubit 4 Fluorometer (Thermo Fisher Scientific, Waltham) and 1 µg total RNA was used for cDNA synthesis. The mRNA was transcribed in cDNA with the High Capacity cDNA Reverse Transcription Kit (Applied Biosystems, #4368814) according the manufacturer’s instructions.

Real-time PCR was performed using the TaqMan® assay (Life Technologies) and TaqMan® primer/probes (Life Technologies) according the manufacturer’s instructions. The RT-PCR was run with Rotor Gene Q (Qiagen, Hilden). The measurement was done as technical triplicates and each C_t_-value was normalized using the housekeeping gene 18 s rRNA. Relative expression levels for each gene were calculated based on comparison with unCRISPRed NUP-MDSC.

The RNA samples that were used for Next Generation RNA-Sequencing were analyzed for RNA integrity with a Bioanalyzer (Agilent Technologies) and then submitted to the European Molecular Biology Laboratory (EMBL, Heidelberg) for further processing. For the analysis undifferentiated NUP cells and MDSCs, co-cultured with 0.1% DMSO, 1 µM retinoic acid or 0.1 µM 3-Deazaneplanocin were used with four biological replicates each.

### Next Generation RNA-Sequencing

The cDNA library for all 16 samples was generated at the European Molecular Biology Laboratory (EMBL). Next Generation RNA-Sequencing was performed with single-end-read (75 bp) sequencing on an Illumina NextSeq. FASTQ files were aligned against the mm10 genome (with Ensembl gene annotations, as acquired from UCSC’s ensGene table in September 2014) using RNA-STAR version 2.5.0b. Quantification at the gene level was done with Subread featureCounts version 1.5.0-p1. FPKMs were then computed in R from the featureCounts output and normalized between samples by scaling each sample by a factor inversely proportional to the geometric mean of the FPKMs in that sample.

Differential expression testing was done with CuffDiff version 2.2.1 using these options “–multi-read-correct–library-type fr-unstranded–dispersion-method pooled” and the full dataset, specifying multiple comparisons (MDSC vs NUP, MDSC + RA vs MDSC, and MDSC + DZNEP vs MDSC).

### Flow Cytometry

Antibody staining was done in presence of Fc receptor blockade (TruStain fcX BioLegend, #101320) in FACS buffer (1× PBS with 1% FBS). SytoxBlue (Thermo Fisher Scientific, S34857) was used for exclusion of dead cells. Antibodies used for flow cytometry were: PerCP-anti-CD11b (Biolegend, #101230), Pacific Blue-anti-CD8 (ebioscience, #48-0081-82). The cells were stained in FACS buffer containing antibodies at 4 °C for 30 min, washed and resuspended in FACS buffer. A FACSAria IIu (BD Biosciences, Heidelberg) and FlowJo software (TreeStar) were used for acquisition and analysis.

### Cytokine measurements

Mouse-specific ELISA for the detection of IFNγ (BioLegend, #430801) and TNFα (Biolegend, #430901) was used to detect cytokines in the supernatants generated *in vitro* according the manufacturer’s instructions.

## Electronic supplementary material

Supplementary table 1

## Data Availability

The datasets generated and analyzed during the current study are available from the corresponding author upon reasonable request.
