# Supplementary material for: Genetic screen in myeloid cells identifies TNF-α autocrine secretion as a factor increasing MDSC suppressive activity via Nos2 up-regulation
Source: Sci Rep. 2018 Sep 7;8:13399. doi: 10.1038/s41598-018-31674-1 (PMC6128861; doi:10.1038/s41598-018-31674-1)
Supplement: Supplementary file 1 — Supplementary table 1 [file 41598_2018_31674_MOESM1_ESM.pdf]

Genetic screen in myeloid cells identifies TNF-α autocrine secretion as a factor increasing MDSC suppressive activity via Nos2 up-regulation

Matthias Schröder, Marit Krötschel, Lena Conrad, Svenja Kerstin Naumann, Christopher Bachran, Alex Rolfe, Viktor Umansky, Laura Helming and Lee Kim Swee

Supplementary Table 1 - Genes screened in the HT assay

Ackr3	Cited1	Ezh2	Irf3	Olr1	Stat6
Ada	Cited2	Fabp4	Irf4	Pdcd1lg2	Tarm1
Adam17	Clec10a	Fam131a	Irf7	Pde5a	Tbc1d8
Adamtsl5	Clec11a	Fcrlb	Irf8	Penk	Tex14
Afmid	Clec4e	Ffar4	Itga2b	Pgf	Tgfb3
Agpat9	Col1a1	Fgf23	Itgax	Phospho1	Tgfbr1
Ahr	Col5a1	Fstl1	Itgb5	Pkm	Tgfbr2
Aldh1l2	Copz2	Gal	Itih1	Pknox2	Tgfbr3
Alox5ap	Cox6a2	Gas6	Jag1	Plekhg1	Thbs1
Anxa2	Cpne6	Gdf15	Jag2	Plxnb2	Timd4
Aqp9	Creg2	Gfi1	Kdr	Pparg	Tlr2
Arg1	Crtac1	Gipr	Klc3	Ptger2	Tnf
Arg2	Crtam	Gpr68	Lcn2	Rarg	Tnfrsf10b
Asb2	Csf1r	Gstt1	Ldhb	Rarres1	Tnfrsf9
Asb4	Csf3	Havcr2	Ldhc	Satb1	Tnfsf15
Atf6	Cx3cr1	Hdac7	Lepr	Serpine1	Traf1
Axl	Cxcl16	Hgfac	Lifr	Slc14a1	Traf2
Cacna1b	Cxcr1	Hif1a	Ltf	Slc16a1	Traf3
Ccl22	Cxcr2	Hk2	Mafb	Slc16a3	Traf6
Ccr2	Cxcr3	Hmgb1	Map2k6	Slc16a7	Trem1
Ccr5	Cxcr4	Hspa1a	Mertk	Slc16a8	Trpm6
Ccr7	Cyba	Hspa1b	Mfge8	Slc1a4	Txn1
Cd200	Cybb	Htra3	Mif	Slc1a5	Txnip
Cd247	Ddah2	Icosl	Mmp12	Slc25a23	Tyro3
Cd24a	Ddit3	Ido1	Mmp14	Slc37a3	Ucp3
Cd274	Ddit4	Ifnlr1	Mmp19	Slc41a2	Wnt6
Cd276	Dlk1	Il10	Mmp8	Slc52a3	Xbp1
Cd300a	Dmkn	Il10ra	Myc	Slc6a9	
Cd36	Dmpk	Il13ra1	Ncf1	Slc7a11	
Cd38	Edn1	Il1b	Ncf2	Slc7a2	
Cd48	Egln3	Il1f6	Ncf4	Slc9a5	
Cd79a	Egr1	Il1r1	Ndrg1	Slfn5	
Cdh1	Egr2	Il3	Nfe2l2	Slirp	
Cdkn2b	Egr3	Il5	Nodal	Smpdl3b	
Cebpb	Eid2	Il6	Nos2	Snai3	
Cebpe	Eif2ak3	Il6st	Nt5e	Snx9	
Cflar	Enpp6	Il7r	Nuak1	Stat1	
Cish	Ern1	Insl6	Oit3	Stat3	
